# Common biological phenotypes characterize the acquisition of platinum-resistance in epithelial ovarian cancer cells

**DOI:** 10.1038/s41598-017-07005-1

**Published:** 2017-08-02

**Authors:** Maura Sonego, Ilenia Pellizzari, Alessandra Dall’Acqua, Eliana Pivetta, Ilaria Lorenzon, Sara Benevol, Riccardo Bomben, Paola Spessotto, Roberto Sorio, Valter Gattei, Barbara Belletti, Monica Schiappacassi, Gustavo Baldassarre

**Affiliations:** 1 Division of Molecular Oncology, National Cancer Institute, 33081 Aviano, Italy; 2 Experimental OncoHematology, National Cancer Institute, 33081 Aviano, Italy; 3 Medical Oncology C CRO Aviano, IRCCS, National Cancer Institute, 33081 Aviano, Italy

## Abstract

Standard of care for Epithelial Ovarian Cancer (EOC) patients relies on platinum-based therapy. However, acquired resistance to platinum occurs frequently and predicts poor prognosis. To understand the mechanisms underlying acquired platinum-resistance, we have generated and characterized three platinum-resistant isogenic EOC cell lines. Resistant cells showed 3-to 5- folds increase in platinum IC50. Cross-resistance to other chemotherapeutic agents commonly used in the treatment of EOC patients was variable and dependent on the cell line utilized. Gene expression profiling (GEP) of coding and non-coding RNAs failed to identify a common signature that could collectively explain the mechanism of resistance. However, we observed that all resistant cell lines displayed a decreased level of DNA platination and a faster repair of damaged DNA. Furthermore, all platinum resistant cell lines displayed a change in their morphology and a higher ability to grown on mesothelium. Overall, we have established and characterized three new models of platinum-resistant EOC cell lines that could be exploited to further dissect the molecular mechanisms underlying acquired resistance to platinum. Our work also suggests that GEP studies alone, at least when performed under basal culture condition, do not represent the optimal way to identify molecular alterations linked to DNA repair pathway defects.

## Introduction

Epithelial ovarian cancer (EOC) is the fourth leading cause of cancer death in women. High mortality rate is mainly due to late diagnosis, when tumours have spread throughout the abdominal cavity in ~75% of the cases^1^. Standard care for these patients combines radical surgery with platinum-taxol chemotherapy^1^. The development of a platinum resistant disease is a frequent event in advanced EOC patients and predicts poor prognosis^1^. The response to first line platinum-based therapy also dictates the subsequent treatment options and EOC patients are clinically classified as platinum refractory, resistant, partially sensitive and sensitive based on the duration of the response to first line therapy^1, 2^.

Molecular and morphological analyses divide EOC in two main subgroups^1, 3, 4^. The largest one comprises high grade EOC that are mostly of serous histotype but could also be of endometrioid or undifferentiated histologies^1^. High grade EOC are characterized by p53 gene mutations, genomic instability, DNA copy number alterations and few other distinct recurrent mutations^1, 5^.

The emergence of platinum-resistant clones under the pressure of chemotherapy hampers treatment efficacy^6^ and relapsed resistant EOCs lack recurrent actionable mutations^7^. Recurrent resistant EOCs almost invariably grow as metastatic disease since the primary tumour is removed during the course of treatment with upfront or interval surgeries^1^. In the majority part of EOC patients, secondary growth locations are peritoneum, omentum and organs located in the peritoneal cavity^8, 9^. Whether and how the acquisition of a platinum resistant phenotype confers also the ability to grow at distant site is still unproved.

Few models of isogenic ovarian cancer platinum resistant cell lines exist. To our knowledge, these models include NOS2, 2008, A2780, COC1, SKOV3, COV362 and COV413 cell lines^10–16^. Recent evidences suggest that some of these models were derived from other cancer and misclassified as ovarian^17^ while others are unlikely to be reliable models of high grade EOC^18, 19^. In particular, the most used A2780 and SKOV3 and their platinum-resistant isogenic cell lines were highly questioned as models of high grade EOC^18, 19^.

High grade serous and endometrioid EOC are the most common histotypes and can also coexist in the same patient. Therefore, setting up these models and studying the molecular mechanisms at the basis of the onset of acquired resistance to platinum in appropriate cellular models, may suggest new possible strategies to overcome resistance and represent a very relevant topic in ovarian cancer research.

## Results

### Generation of cisplatin-resistant cells

We selected KURAMOCHI and OVSAHO as models of high grade serous- and MDAH-2774 (hereafter referred as MDAH) and TOV-112D as models of high grade endometrioid-carcinomas, based on published results^18, 20, 21^. All of the four cell lines carried mutations in TP53 and two of them, KURAMOCHI and MDAH, also in BRCA2 gene (Supplementary Figure S1A). Although, OVSAHO cells have been reported to carry a homozygous deletion of BRCA2^18^, we could not detect it by our sequence analysis.

First, we treated cells with increasing concentration of cisplatin for 72 hours and established that the cisplatin concentration to achieve 50% of cell death (IC50) of the different cell lines ranged between 2 and 5 µM (Supplementary Figure S1B). Therefore, all these cell lines can be considered cisplatin-sensitive.

**Figure 1:**
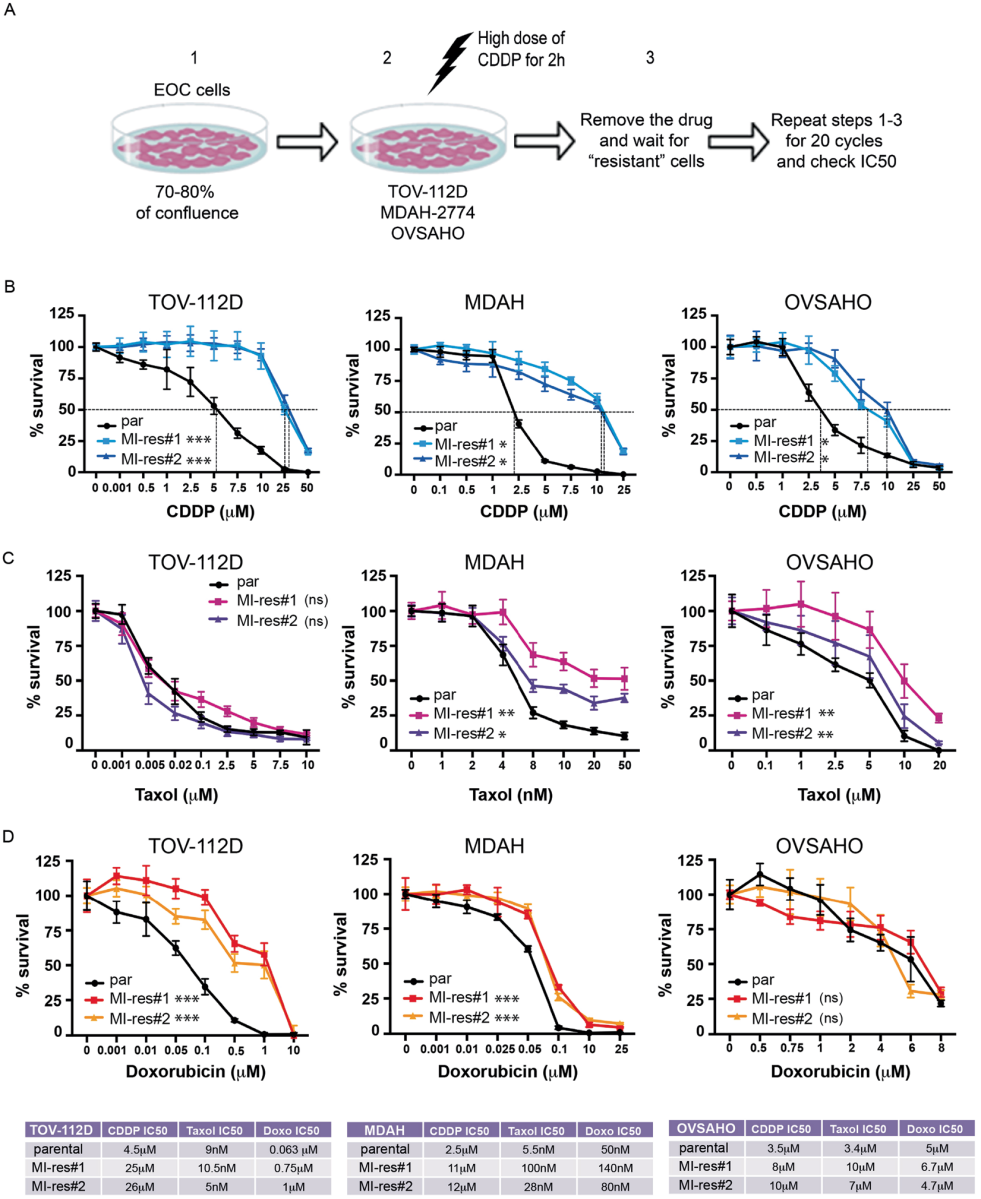
Generation of cisplatin-resistant EOC cells. (**A**) Experimental design used in the generation of cisplatin-resistant (MI-res) cells. Cells, plated at high confluence (1), were exposed for 2 hours to a cisplatin dose 10-fold higher than their calculated IC50 (2). After a suspension period (3), recovered cells were subjected again to step 1–3, for additional 20 cycles and then their cisplatin sensitivity was assessed again. (**B–D**) Cisplatin (**B**), Taxol (**C**) and Doxorubicin (**D**) dose response curves using parental and MI-res cells, as indicated. Results are expressed as percentage of viable cells respect to untreated cells. IC50 (half maximal inhibitory concentrations) are reported (n = 3 biological replicates each performed in triplicate). In the lower table, the IC50 calculated for each drug and cell line is shown. In (**B**,**C** and **D**), the difference between parental cells and each MI-res clone is reported. Statistical significance was determined by repeated-measures analysis of one-way-ANOVA test. *p < 0.05; **p < 0.01; ***p < 0.001; ns = not significant.

Cisplatin-resistant EOC cells were generated using the pulse method (Fig. 1A), that is considered the most appropriate strategy to generate drug-resistant ovarian cancer cells *in vitro* ^22^. However, by this approach we were unable to select cisplatin-resistant KURAMOCHI cells, in line with what already observed for another model of high grade serous EOC, COV362 cells^10, 18^. Therefore, we excluded the KURAMOCHI cell line from this study.

By performing 20 cycles of cisplatin pulse treatment, we selected two cisplatin-resistant populations from each cell line (hereafter named MI-res) (Fig. 1A). MI-res cells, displayed a cisplatin IC50 3- to 5-fold higher than parental cells (Fig. 1B). Using TOV-112D as model, we experimentally verified that the resistant phenotype was stable and maintained for at least two months, independently from the presence of cisplatin in the culture medium (Supplementary Figure S1C).

We next tested if MI-res cells acquired cross-resistance to Taxol and Doxorubicin, two other chemotherapeutic drugs commonly used to treat EOC patients. MDAH and OVSAHO, but not TOV-112D, MI-res cells displayed an IC50 for Taxol of 2- to 5-fold higher than parental cells (Fig. 1C). In the case of Doxorubicin, MDAH and TOV-112D, but not OVSAHO, MI-res cells were more resistant than parental cells (Fig. 1D).

### Cell cycle progression of cisplatin-resistant cells

**Figure 2:**
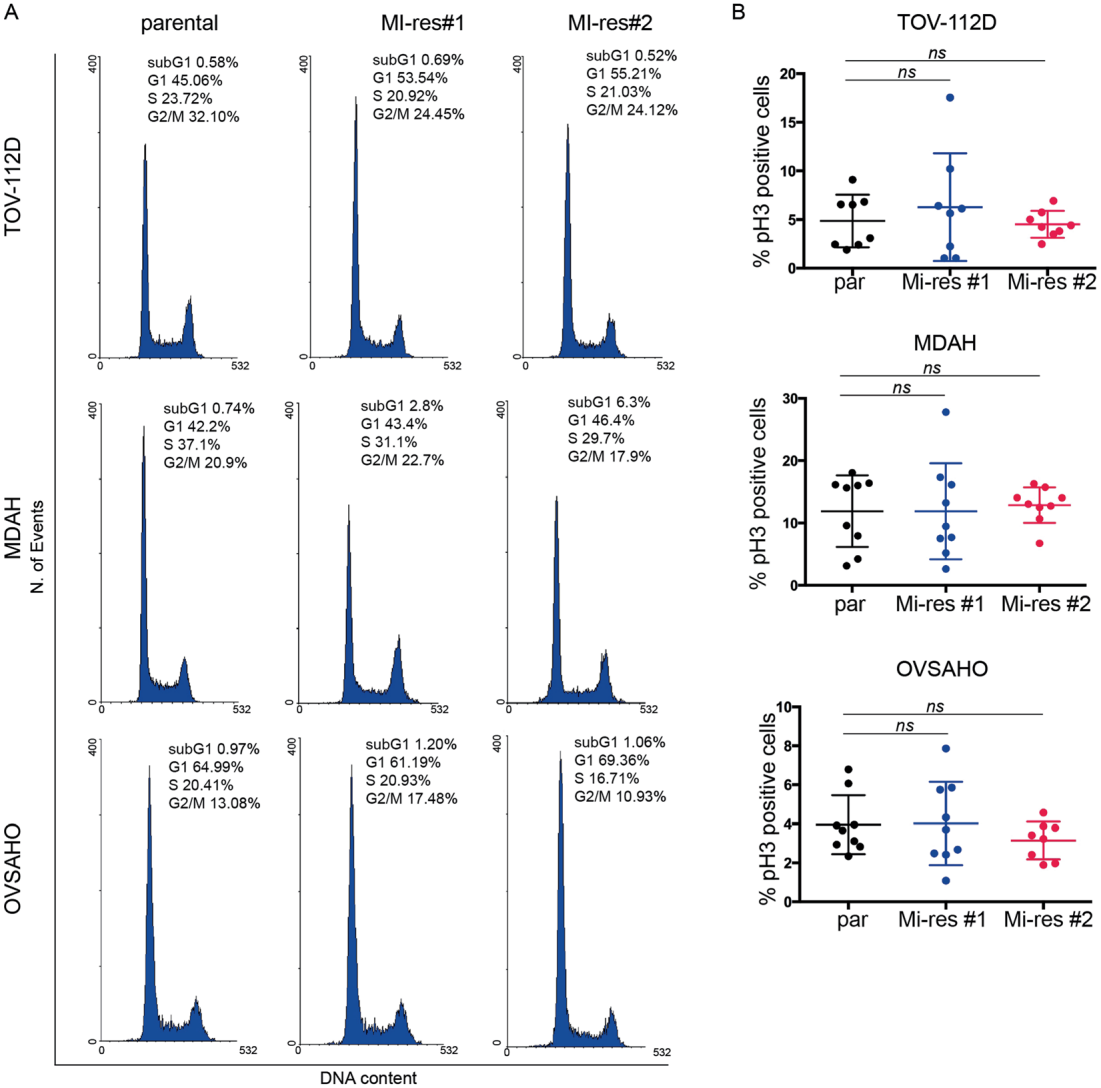
MI-res cells show a similar cell cycle progression. (**A**) FACS analyses of DNA content of parental and MI-res cells in exponentially growing conditions. The cell cycle distribution is reported in each plot. A representative experiment is shown. (**B**) Evaluation of mitotic index of parental and MI-res cells in exponentially growing conditions. Mitotic index was calculated by immunofluorescence analyses, using the expression of pS10-H3 as a marker of mitotic cells. Data are expressed as percentage of pH3-positive cells. Statistical significance was determined by a two-tailed, unpaired Student’s t-test (ns = not significant).

We next checked if platinum resistance was linked to an altered cell cycle progression. Under basal culture condition, FACS analyses of DNA content did not highlight any significant variation in cell cycle distribution (Fig. 2A). This result was confirmed by immunofluorescence analyses of phosphorylated Histone H3 (marker of mitotic cells), showing no differences in the mitotic index of parental and MI-res cells (Fig. 2B). Accordingly, the expression of Ki67, Cyclin E and Cyclin A, also used as markers of proliferating cells, did not significantly differ between parental and MI-res cells (Supplementary Figure S2).

### Morphology and adhesion ability of cisplatin-resistant cells

**Figure 3:**
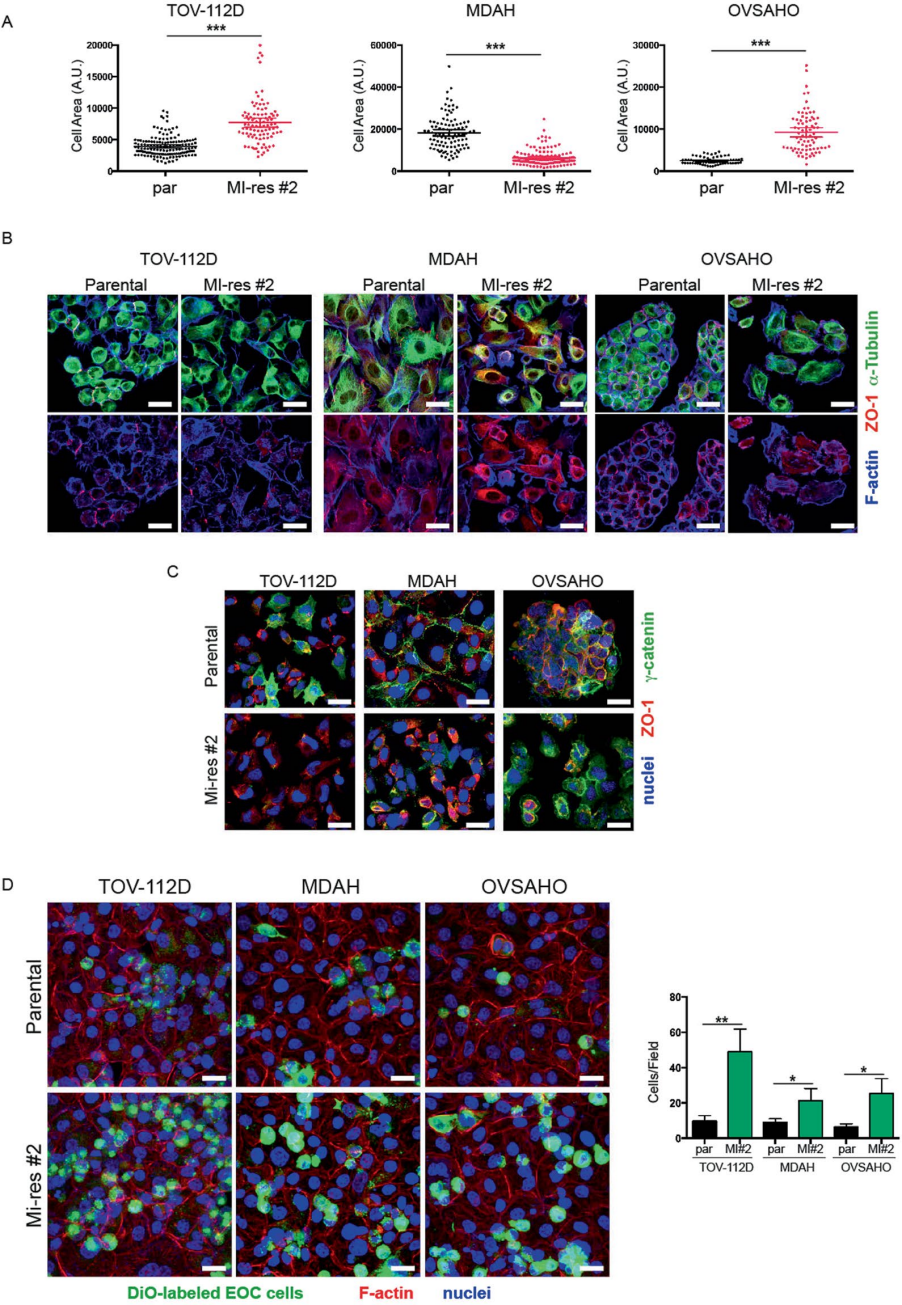
MI-res cells show altered cell morphology. (**A**) Dot plot showing the area of parental and MI-res cells in exponentially growing conditions. Data were obtained using the ImageJ software and each dot corresponds to one cell. At least 80 cells were scored for each cell line. Statistical significance was determined by a two-tailed, unpaired Student’s t-test (***p < 0.001). (**B**) Representative images of parental and MI-res cells, immunostained for the expression and localization of α-tubulin (green), ZO-1 (red) and F-Actin (blue), are reported. Scale bar = 25 µm. (**C**) Representative images of γ-catenin (green) and ZO-1 (red) in parental and MI-res cells cultured in exponentially growing conditions. Nuclei are in blue. Scale bar = 25 µm. (**D**) Maximal projections of images of parental and MI-res cells labeled with the green fluorescent marker DiO (green) and cultured on a monolayer of mesothelial cells for 24 hours. Cells were then fixed and stained with Phalloidin (F-Actin, red) and TO-PRO3 (nuclei, blue). In the right graph, the number (mean ± SD) of cancer cells/field is reported. Statistical significance was determined by a two-tailed, unpaired Student’s t-test (*p < 0.05 and **p < 0.01) (MI#2 = MI-res#2).

A characteristic shared among all MI-res cells was an evident alteration of their morphology respect to the one observed in matched parental cells (Fig. 3 and Supplementary Figure S3). Parental TOV-112D cells are small and comprise both rounded and spindled-shaped cells, while the correspondent MI-res cells were frankly polygonal with a significantly increased cell area. Conversely, MDAH cells are large epithelial-like cells and the correspondent resistant clones displayed a significantly smaller area. Finally, OVSAHO parental cells are small round-shaped cells growing in small epithelial-like islets, while OVSAHO MI-res cells were larger and more elongated (Fig. 3A and Supplementary Figure S3A). These morphological modifications suggested that the balance between epithelial and mesenchymal status was altered in MI-res cells, possibly through alteration of the Epithelial-Mesenchymal Transition (EMT) and/or Mesenchymal-Epithelial Transition (MET) processes. It has been proposed in EOC that an EMT-like process, characterized by alternation of EMT and MET, could confer the ability to spread, attach and grow in the peritoneal lining and, eventually, favour the appearance of chemoresistant clones^23^. To verify this possibility, we tested the expression of known modulator of EMT and MET, namely SNAIL, SLUG, TWIST-1 and ZEB1, by Western Blot analyses. The results of our analyses showed a change of at least one of these transcription factors in MI-res cells (Supplementary Figure S3B), suggesting that an altered balance between epithelial and mesenchymal status could be a common feature of platinum resistant cells. In accord with these evidences, we observed that all MI-res cells commonly avoided the growth in aggregates, also when plated at high density (Fig. 3B). Immunofluorescence analyses evaluating the expression and distribution of F-actin, α-tubulin, ZO-1 (marker of tight junctions) and γ-catenin (marker of adherens junctions), confirmed that MI-res cells displayed a different organization of actin and microtubular cytoskeleton, with respect to their parental counterparts (Fig. 3B). Moreover, MI-res cells failed to form well-organized cell-cell junctions, as demonstrated by ZO-1 and γ-catenin redistribution in the cytosol rather than at the cell membrane and by the fact that ZO-1 and γ-catenin failed to properly locate at the cell-cell contacts as observed in parental cells (Fig. 3C and Supplementary Figure S4).

Mesothelial cells represent the cellular component in which platinum-resistant EOC cells grow *in vivo*, to spread from primary lesions. We thus tested the growth of parental and MI-res cells on a mesothelial cell layer. Ovarian cells, either parental or MI-res, were labeled with the lipophilic green fluorescent dye, DiO, and seeded on a mesothelial cell layer for 24 hours. All MI-res cells adhered and grew on the mesothelium at higher rate than the parental ones, as demonstrated by immunoflurescence analyses followed by three-dimensional reconstruction of confocal images (Fig. 3D and Supplementary Figure S5).

### Response to cisplatin-induced damage of MI-res cells

**Figure 4:**
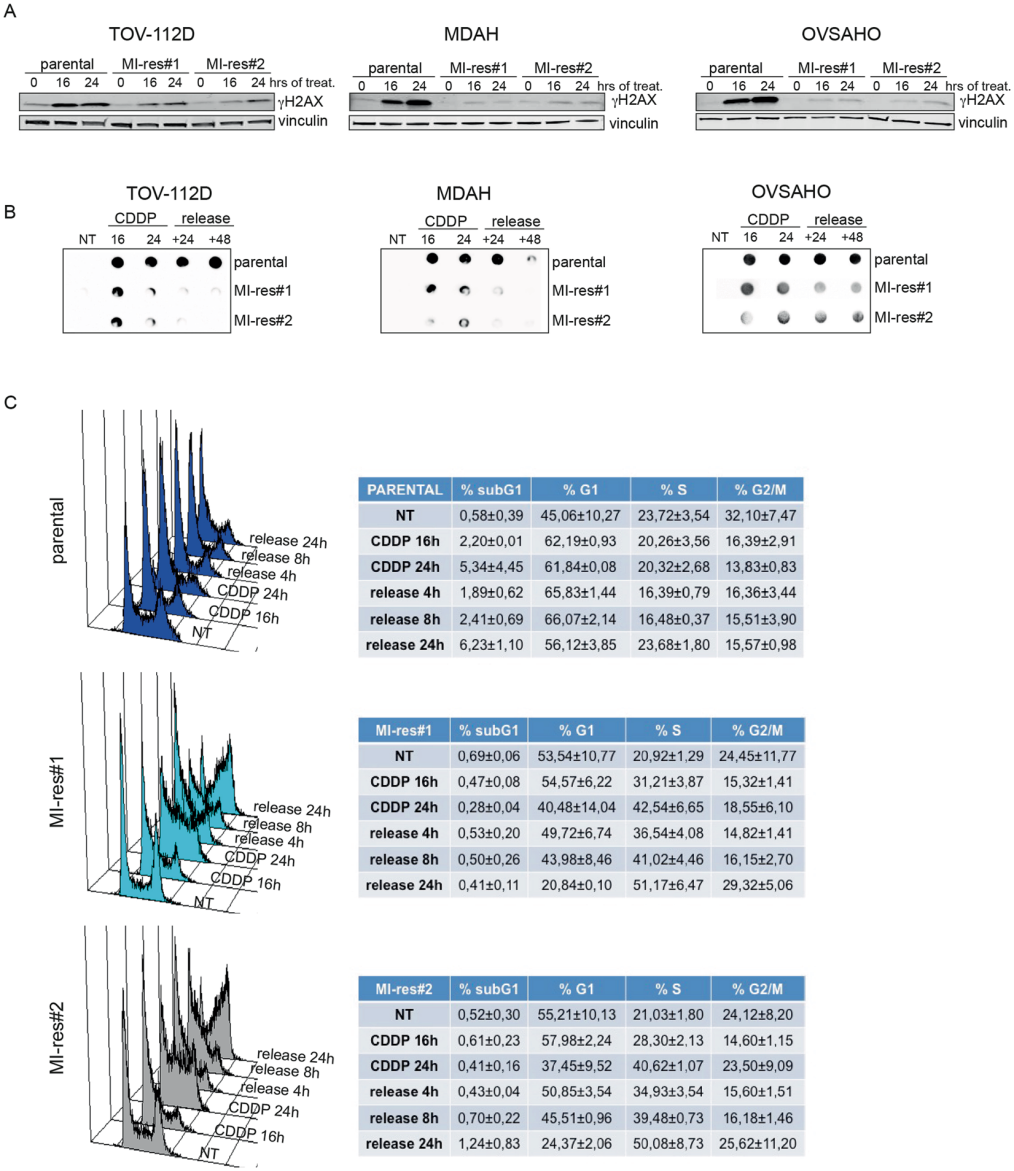
MI-res cells accumulate less cisplatin-induced DNA damage respect to parental cells. (**A**) Western blot analyses of S139 phosphorylated Histone H2AX (γH2AX) expression, used as marker of DNA damage, in parental and MI-res cells untreated (0) or treated with cisplatin for 16 and 24 hours, as indicated. Vinculin was used as loading control. (**B**) Dot blot analyses evaluating the amount of platinated-DNA in parental and MI-res cells untreated (NT) or treated with cisplatin for 16 and 24 hours and then released in cisplatin-free medium for additional 24 (+24) or 48 (+48) hours to allow for DNA repair. (**C**) FACS analyses of DNA content in TOV-112D parental and MI-res cells untreated (NT) or treated with cisplatin for 16 and 24 hours and then released in cisplatin-free medium for additional 4, 8 or 24 hours, as indicated. A typical histogram for each cell line is shown and the correspondent cell cycle distribution (mean ± SD n = 3 biological replicates) is reported in the lower tables.

Next, we investigated the response of MI-res cells to cisplatin. Following cisplatin treatment for 16 or 24 hours, all MI-res cells accumulated less DNA damage, as demonstrated by the phosphorylation levels of Histone H2AX (γH2AX). Moreover, we could detect a reduced DNA platination and a faster removal of platinum from the DNA that paralleled the reduced expression of γH2AX (Fig. 4A and B). Accordingly, MI-res cells resolved the cisplatin-induced S phase block within 4–8 hours from cisplatin release, while parental cells accumulated in G1 and started to re-enter the cell cycle only 24 hours after cisplatin release (Fig. 4C and Supplementary Figure S3).

### Cell-specific alterations of cisplatin transporters are present in MI-res cells

**Figure 5:**
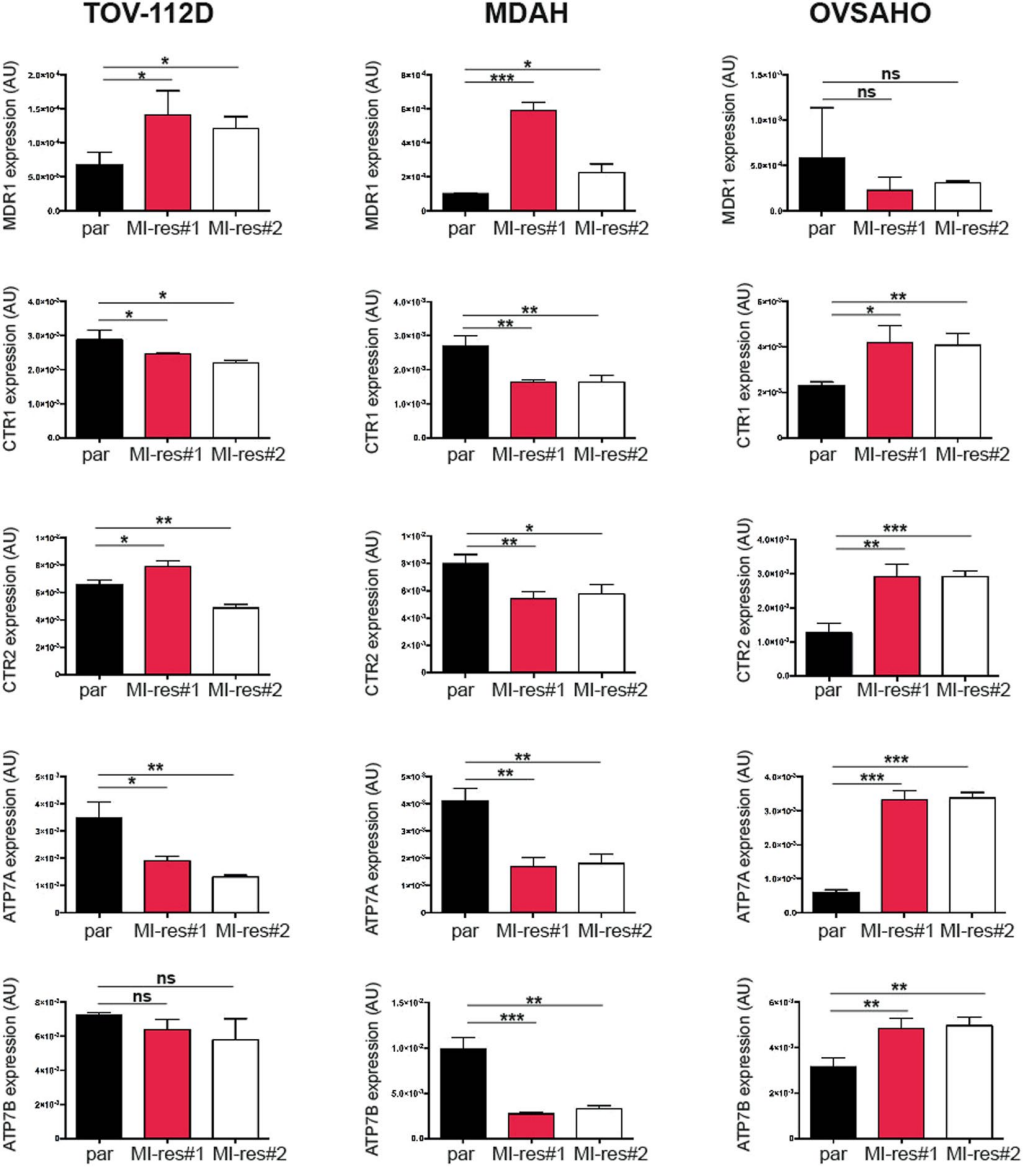
Expression of cisplatin-transporters in parental and MI-res cells. Graphs showing the expression of MDR1, CTR1, CTR2, ATP-7A and ATP-7B cisplatin-transporters in the indicated cell lines, evaluated by qRT-PCR analyses. mRNA levels was analyzed in duplicate and normalized using SDHA housekeeping gene. Data represent the mean ± SD of 3 biological replicates, performed in duplicate. Statistical significance was determined by a two-tailed, unpaired Student’s t-test (ns = Not Significant, *p < 0.05, **p < 0.01, ***p < 0.001).

The above data on DNA platination suggested that MI-res could have acquired defects in the uptake and/or efflux of cisplatin. Decreased cisplatin influx is mainly mediated by the copper transporters CTR1, while increased cisplatin efflux is mediated mainly by MDR1, ATP7A and ATP7B transporters^24^. Using qRT-PCR analyses we observed that all MI-res cells displayed a deregulated expression of CTR1, CTR2, MDR1, and/or ATP-7A, although not in a common way. A significant decreased expression of CTR1, that could explain a decreased cisplatin influx, was observed in MDAH and TOV-112D MI-res respect to parental cells. These cells also upregulated the expression of MDR1 gene that promotes the efflux of cisplatin. OVSAHO MI-res cells expressed similar amount of MDR1 and strongly increased expression of ATP-7A, ATP-7B respect to parental cells (Fig. 5). CTR2 is a copper uptake protein with substantial structural homology to CTR1, but it plays an opposite role in the transport of platinum drugs. CTR2 inhibits cisplatin accumulation in the cell and its expression has been associated to increased platinum-resistance in ovarian cancer^25^. Our results in OVSAHO cells were in accordance with these data, while TOV-112D and MDAH cells, that more likely rely on the overexpression of MDR1 and the downregulation of CTR1, did not display significant and univocal variation in CTR2. Overall these data suggest that all MI-res clones commonly displayed a deregulation in platinum transport that could be due to different molecular alteration specific for each cell line.

### Gene expression profiling failed to identify signalling pathways commonly altered in MI-res cells

**Figure 6:**
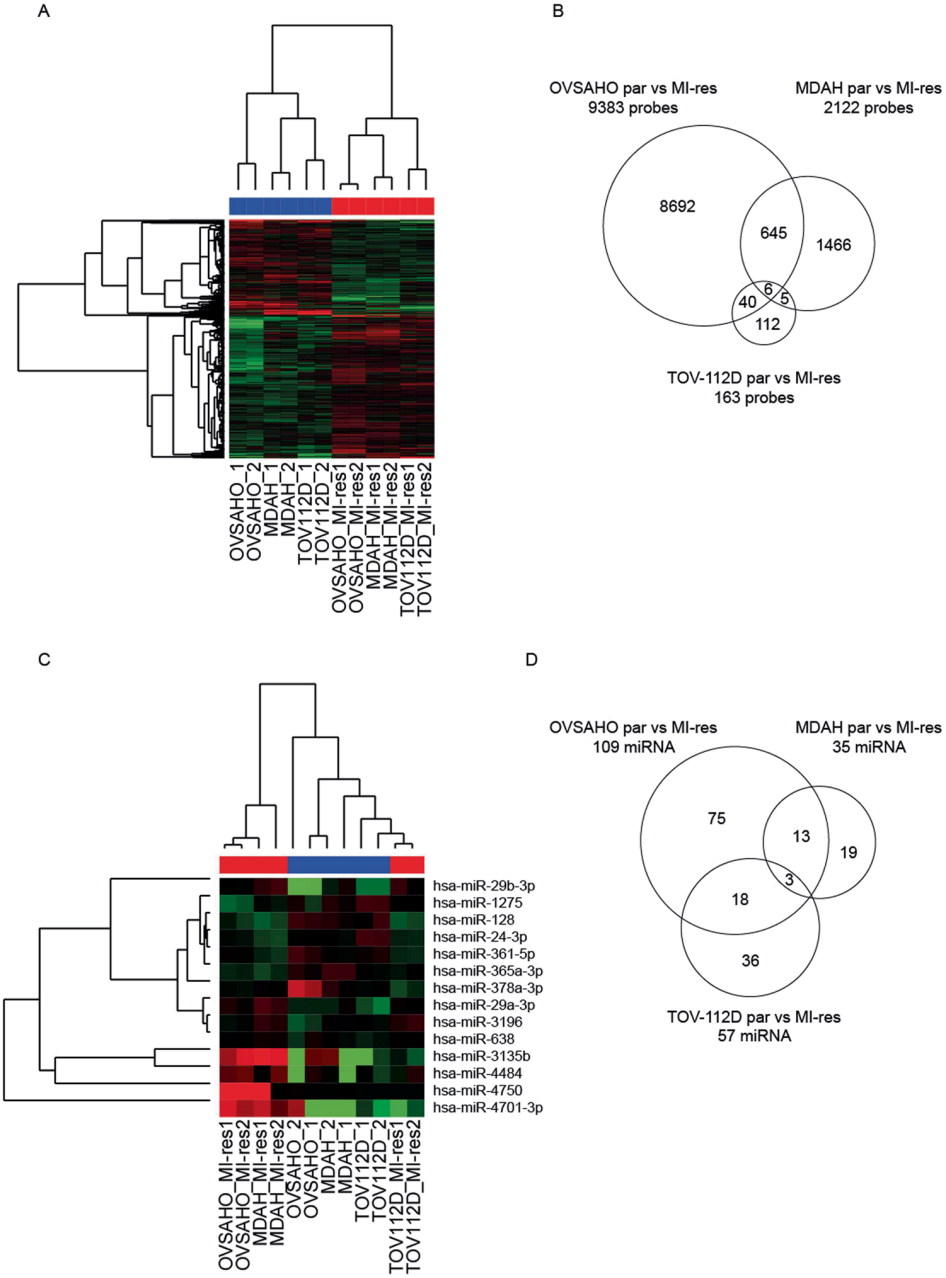
Expression of coding and non-coding genes does not identify common gene expression alteration in MI-res cells. (**A**) Heat map of supervised clustering analyses evaluating the expression of coding and long non-coding RNAs in parental and MI-res cells, as indicated. (**B**) Venn diagram showing the genes differentially expressed between parental and MI-res cells, in each cell line. Few genes were commonly altered in the three models and none in the same direction (*i.e*. always increased or decreased in MI-res *vs* parental cells). (**C**) Heat map of supervised clustering analyses evaluating the expression of microRNAs in parental and MI-res cells, as indicated. (**D**) Venn diagram showing the microRNAs differentially expressed between parental and MI-res cells in each cell line. Only 3 microRNAs were commonly altered in the three models and none in the same direction (*i.e*. always increased or decreased in MI-res *vs* parental cells). For both coding and non coding gene expression analyses, 2 samples for each parental and 2 for each MI-res cell line were analyzed.

Major alterations that we could observe in MI-res cells impacted on the regulation of cell morphology (Fig. 3) and on the response to DNA damage (Fig. 4), but we could not identify any common molecular mediator(s) of these changes. We thus used a genome wide gene expression profiling (GEP) approach to identify common molecular alterations and/or altered pathways explaining the phenotypes observed. However, unsupervised clustering analyses clearly showed that MI-res cells did not cluster together but remained similar to their parental cells, in term of mRNA, long non-coding RNA and microRNAs expression (Supplementary Figure S4A and B). Supervised clustering also failed to identify genes and microRNAs commonly and significantly modified in the cisplatin-resistant models (Fig. 6A–D). Moreover, gene set enrichment analyses, comparing MI-res with parental cells again failed to identify pathways commonly altered in the three models, showing that MDAH MI-res cells were enriched for pathways related to cell cycle regulation, TOV-112D MI-res cells for pathways related to immune response and vesicular trafficking while OVSAHO MI-res cells showed few significantly enriched pathways (Table 1).

**Table 1:** Gene set up-regulated in EOC cisplatin-resistant *versus* parental cells according to Gene Set Enrichment Analysis (GSEA).

NAME OF GENE SET	Genes in the gene set	Enrichment Score	Normalized Enrich. Score	False Discovery Rate q-value
GO_REGULATION_OF_TRANSCRIPTION_INVOLVED_IN_G1_S_TRANSITION_OF_MITOTIC_CELL_CYCLE	24	−0,6534601	−21.508.212	0,042720232
GO_NUCLEAR_NUCLEOSOME	36	−0,5882183	−21.454.525	0,021360116
GO_SISTER_CHROMATID_COHESION	104	−0,48625126	−2.145.334	0,014240078
GO_PROTEASOME_REGUL_PARTICLE_BASE_SUBCOMPLEX	12	−0,77284837	−2.117.518	0,016993934
GO_SISTER_CHROMATID_SEGREGATION	161	−0,42489344	−20.574.353	0,037104394
GO_REGULATION_OF_MRNA_3_END_PROCESSING	26	−0,62627596	−20.521.066	0,032315142
**TOV-112D**				
**NAME OF GENE SET**	**Genes in the gene set**	**Enrichment Score**	**Normalized Enrich. Score**	**False Discovery Rate q-value**
GO_INTERFERON_GAMMA_MEDIATED_SIGNAL_PATHWAY	51	−0,73018473	−27.059.295	0
GO_RESPONSE_TO_TYPE_I_INTERFERON	50	−0,7179578	−26.159.458	0
GO_CELLULAR_RESPONSE_TO_INTERFERON_GAMMA	80	−0,64604324	−24.847.577	0
GO_RESPONSE_TO_INTERFERON_GAMMA	98	−0,5872227	−2.320.558	0
GO_NEGATIVE_REGULATION_OF_VIRAL_PROCESS	75	−0,6012249	−23.173.413	0
GO_DEFENSE_RESPONSE_TO_VIRUS	121	−0,5367214	−23.013.668	0
GO_ANTIGEN_PROCESSING_AND_PRESENTATION_OF_ENDOGENOUS_PEPTIDE_ANTIGEN	11	−0,8918182	−21.674.275	0,001130333
GO_LYSOSOMAL_LUMEN	72	−0,5250776	−21.658.518	9,89E + 02
GO_MHC_PROTEIN_COMPLEX	20	−0,7326118	−21.546.667	8,79E + 01
GO_NEG_REGULATION_OF_VIRAL_GENOME_REPLICATION	42	−0,615934	−2.118.308	0,001588091
GO_LUMENAL_SIDE_OF_MEMBRANE	25	−0,68945616	−21.111.588	0,002159565
GO_CYTOKINE_MEDIATED_SIGNALING_PATHWAY	295	−0,4352455	−21.057.432	0,001979601
GO_ER_TO_GOLGI_TRANSPORT_VESICLE_MEMBRANE	45	−0,6056494	−21.049.874	0,001827324
GO_INNATE_IMMUNE_RESPONSE	362	−0,42982835	−20.899.904	0,00226092
GO_PHAGOSOME_MATURATION	33	−0,5923688	−20.738.387	0,003704131
GO_ANTIGEN_PROCESSING_AND_PRESENTATION_OF_PEPTIDE_ANTIGEN_VIA_MHC_CLASS_I	79	−0,5064992	−2.066.589	0,00494183
GO_ANTIGEN_PROCESSING_AND_PRESENTATION_OF_ENDOGENOUS_ANTIGEN	12	−0,86477375	−20.513.408	0,006497909
GO_VACUOLAR_LUMEN	90	−0,49934855	−20.206.945	0,009655423
GO_PHAGOSOME_ACIDIFICATION	22	−0,6625092	−2.016.892	0,01041846
GO_NEGATIVE_REGULATION_OF_MULTI_ORGANISM_PROCESS	109	−0,5047006	−20.165.906	0,009897537
GO_RESPONSE_TO_VIRUS	189	−0,45580903	−2.012.423	0,010178529
GO_DNA_SYNTHESIS_INVOLVED_IN_DNA_REPAIR	69	−0,53206104	−19.991.966	0,011879611
GO_ANTIGEN_PROCESSING_AND_PRESENTATION_OF_EXOGENOUS_PEPTIDE_ANTIGEN_VIA_MHC_CLASS_I	57	−0,49659282	−19.854.096	0,01581375
GO_ER_TO_GOLGI_TRANSPORT_VESICLE	59	−0,51620644	−19.475.952	0,025982931
**OVSAHO**				
**NAME OF GENE SET**	**Genes in the gene set**	**Enrichment Score**	**Normalized Enrich. Score**	**False Discovery Rate q-value**
GO_LIGAND_GATED_CALCIUM_CHANNEL_ACTIVITY	14	−0,7387153	−21.681.046	0,047560018
GO_MHC_PROTEIN_COMPLEX	23	−0,63676775	−21.423.683	0,038119253
GO_MRNA_SPLICE_SITE_SELECTION	26	−0,59817517	−20.946.863	0,047710102
GO_LIGAND_GATED_CALCIUM_CHANNEL_ACTIVITY	14	−0,7387153	−21.681.046	0,047560018
GO_MHC_PROTEIN_COMPLEX	23	−0,63676775	−21.423.683	0,038119253

## Discussion

Here, we report the molecular and biological characterization of three isogenic cisplatin-resistant cell lines derived from high grade EOC. To our knowledge this is the first report comparing three different EOC models of acquired cisplatin-resistance. This approach allowed us to define that, although common phenotypic alterations exist in resistant cells, different molecular alteration underline the acquired resistance of high grade EOC.

Several considerations arise from our study that can also be relevant for future studies on platinum-resistance. The first observation concerns the common ability of cisplatin-resistant cells to resolve the platinum-induced DNA damage (Fig. 4), suggesting that drug uptake, detoxification and excretion along with the DNA repair pathway play a central role in the instauration of a resistant phenotype. These data are in accord with the frequent overexpression of the ABCB1 gene (encoding for MDR1) in resistant but not in primary EOC samples, possibly due to the rearrangement of promoter sequences as recently reported in the comprehensive genomic characterization of resistant EOC^7^.

Our gene set enrichment analyses failed to identify this, or other related, pathway as significantly changed in MI-res respect to parental cells when analyzed under basal culture condition. A possible explanation for this unexpected result could be that the differences between parental and resistant cells could become evident only under the pressure of platinum treatment or that post-translational modification are the ones principally involved in the acquisition of the higher ability of MI-res cells to resolve the platinum induced DNA damage. Accordingly, a recent proteomic characterization of high grade serous ovarian cancer suggests that, despite the complexity of their genomic alterations, a functional convergence on a subset of key signal transduction pathways exists in these tumours^26^. Another intriguing point suggested by our data is that acquired resistance *in vivo* could be quite different from the one selected *in vitro*. Thus, models such as the PEO1/PEO4/PEO6 cells, that derive from the same patient at different stages of disease, could represent an extremely relevant and valuable tool to understand the mechanisms underlying platinum resistance and to identify new possible ways of intervention^27, 28^. Altogether, literature data and our results together suggest that acquired platinum-resistance in ovarian cancer is due to multiple genetic and epigenetic alterations that should be recapitulated *in vitro* under the pressure of specific treatments (e.g. platinum). Future experiments are necessary to answer to this question.

We observed that cross-resistance to doxorubicin, that also induce a DNA damage response^29^, was cell-type specific (Fig. 1). Identifying biomarkers of cross-resistance, to avoid unnecessary treatments to platinum resistant patients, would result in relevant clinical benefit for patients. There is an interesting clinical debate on the use of platinum-free interval, in the clinical settings of platinum-sensitive ovarian cancer patients, to exploit valid chemotherapeutic alternative^2^. Our data, showing that, at least *in vitro*, the acquired resistant phenotype is maintained regardless to presence or absence of platinum, seems to support the hypothesis that the use of platinum-free interval could be considered a therapeutic strategy only in platinum-sensitive ovarian cancer.

An evident morphological change accompanied the acquisition of the resistant phenotype. This included the reorganization of cytoskeleton, cell-cell junctions and adhesion abilities (to mesothelium monolayer) (Fig. 3) suggesting that EMT and MET processes could be linked to a different response to platinum in ovarian cancer, as recently proposed by others^30, 31^. We detected a different expression of SNAIL, TWIST1 and ZEB1 in our resistant cell clones, supporting the notion that the resistant phenotype is accompanied by an induction of an EMT-like process. Also in this setting, it is predictable that the differences between parental and MI-res cells will be better evident under the pressure of platinum or in other stress conditions. The finding that all MI-res cells display increased adhesion and growth on the mesothelium, along with the presence of an altered cell-cell junction, encourage us to further investigate the mechanisms responsible for the increased ability to disseminate in the peritoneal cavity and to adhere at distant sites as key and common properties of platinum resistant cells. This evidence merits to be better studied at molecular level in the future with proteomic and/or functional studies to identify possible key molecules linking platinum-resistance to cell plasticity.

## Methods

### Drugs

Cisplatin was purchased from TEVA Italia, Paclitaxel (TAXOL®) from ACTAVIS (Dublin, Ireland) and Doxorubicin from Ebewe Italia.

### Cell culture

MDAH-2774 (CRL-10303), TOV-112D (CRL-11731) cells were from ATCC while OVSAHO (JCRB1046) and KURAMOCHI (JCRB0098) cells were from JCRB Cell Bank. All cell lines were maintained in RPMI-1640 medium (Sigma–Aldrich Co.) containing 10% heat-inactivated FBS, 100 U/ml penicillin and streptomycin (complete medium) at 37 °C in a 5% CO_2_ atmosphere. Cisplatin-resistant (MI-res) isogenic cells were generated by treating EOC parental cells for growth for 2 hours with a cisplatin dose 10-fold higher than the calculated IC50 and then allowing to re-grow in drug-free complete medium (pulse treatment). The subsequent drug treatment was administrated when the cells reached again a 70–80% of confluence. In total MI-res cells received 20 pulse treatments. All the experiments were performed with cells kept in cisplatin-free medium for ≥2 months unless otherwise stated.

### Cell viability assay

Cell viability was performed essentially as described previously^32^. Briefly, EOC cells were seeded in 96-well plates and treated with increasing doses of cisplatin, Taxol or Doxorubicin for 72 hours. Cell viability was determined using the CellTiter 96 AQueous kit (Promega). Absorbance was detected at 492 nm using a microplate reader (Infinite® M1000 Pro, Tecan).

### Cell cycle analysis by flow cytometry

EOC cells were plated in 60-mm dishes (8 × 10^5^ cells/dish) and after 24 hours treated with cisplatin. Cells were harvested at the indicated time points, washed with phosphate-buffered saline (PBS), fixed in ice-cold 70% ethanol, and stored at −20 °C overnight. Fixed cells were then washed in PBS and re-suspended in propidium iodide (PI) staining solution (50 μg/ml PI + 100 μg/ml RNaseA, in PBS). Stained cells were subjected to flow cytometry analysis with a FACScan or a FACSCalibur instrument (BD Biosciences). Distribution of cells in G1, S and G2/M phases of the cell cycle was calculated using the WinMDI2.9 software as described^33^.

### Preparation of Cell lysates and Immunoblotting

Cell lysates were prepared using cold RIPA lysis buffer (150 mM NaCl, 50 mM Tris HCl [pH 8], 1% NP40, 0.5% sodium deoxycholate, 0.1% SDS) plus a protease inhibitor cocktail (Complete, Roche), 1 mM NaOV_4_, and 1 mM DTT. Immunoblotting were then performed as reported^32^ using the following primary antibodies: anti-phospho-histone H2AX-S139 (γH2AX) (EMD Millipore), -Ki67 (Abcam), -cyclin D1 (Calbiochem), -cyclin D2, -cyclin D3 (Cell Signalling), -cyclin E, cyclin A - vinculin (Santa Cruz), -RB1, -p27^Kip1^ and -GRB2 (BD Transduction Laboratories).

### Cisplatin-DNA (Pt-DNA) adduct detection by Dot-blot

To detect cisplatin-DNA adducts, genomic DNA was isolated using the Maxwell® DNA-purification kit (Promega) from cells treated as indicated. DNA (500 ng) was denatured at 100 °C for 10 min and spotted onto nitrocellulose membranes (Hybond C; Amersham) using a dot-blot apparatus (Easy-Titer® ELIFA, Pierce). The membranes were then baked for 2 hours at 80 °C and subjected to standard Immunoblot assay using an anti-cisplatin-DNA Adducts (1:500) antibody (EMD Milipore, clone ICR4).

### EOC adhesion on mesothelial cells and Immunofluorescence Analyses

Mesothelial cells (derived from pleural lavage) were seeded on glass coverslips and allowed to grow until they reached complete confluence. 1.5 × 10^5^ EOC cells detached with 5 mM EDTA, labeled with vital green fluorescent lipophilic tracer DiO (Invitrogen) and washed with PBS were then plated on mesothelial layer in serum free medium for 24 hours.

For immunofluorescence analyses cells plated on coverslips were fixed in PBS-4% paraformaldehyde (PFA) at room temperature (RT), blocked in PBS-1% bovine serum albumin (BSA) and stained as indicated with primary antibodies: anti-α-tubulin-fluorescein isothiocyanate (FITC) (Sigma), phospho-Histone H3 (S10) (Upstate), ZO-1 (Cell Signalling) and γ-catenin (BD Biosciences). Then samples were washed in PBS and incubated with secondary antibodies (Alexa-Fluor 488-, 633- or 546-conjugated anti-mouse or anti-rabbit antibodies; Invitrogen) for 1 hour at RT. PI (5 μg/ml + RNaseA) or TO-PRO-3 iodide (Invitrogen) were used to visualize nuclei and Alexa-Fluor 647- or 546-Phalloidin (Invitrogen) for F-actin staining. Coverslips were mounted with glycerol/0.25% DABCO and analyzed using a the Leica Time Lapse AF6000LX workstation, the TCS-SP2 or the TCS-SP8 Confocal Systems (Leica Microsystems Heidelberg GmbH) interfaced with the Leica Confocal Software (LCS) or the Leica Application Suite (LAS) software.

### qRT-PCR

Total RNA was isolated with TRIzol Reagent (Life Technologies). 2 µg of total RNA was retro-transcribed using random hexamers and the AMV Reverse Transcriptase (Promega). 1/20 of the obtained cDNAs was amplified using primers described in Supplementary Table S1. Standard curves (10-fold dilution from 10^−1^ to 10^−4^ attomoles) were prepared and analyzed by qRT-PCR using the MyiQ2 Two Color Real-time PCR Detection System (Biorad) as described^34^.

### Gene and miR expression profiling and data mining tools

Gene and miR expression profiling were performed essentially as described^33, 35, 36^. Briefly, single-color hybridization microarray experiments for miRome were performed with 100 ng total RNA/sample labeled with Cyanine(Cy)−3 dye using the microRNA Complete Labeling System & Hyb Kit (Agilent Technologies). Cy3-labeled RNA was hybridized to the Human microRNA microarray Version 3 (8 × 60 K) from the Sanger database v12.0 (Agilent Technologies). GEP was performed using the Whole Human Genome (8 × 60 K) oligo microarray platform (Agilent Technologies). Slides were analyzed by the Agilent Microarray Scanner, using the Agilent Feature Extraction Software 10.7.3 (Agilent Technologies). Pre-processing steps have involved quality check, exponential-normal convolution background subtraction, lowess and quantile normalization. After pre-processing, prefiltering was applied aimed at eliminating all genes whose interquartile range was below the 20th percentile of distribution (genes whose expression was overall not differentially modulated).

After pre-processing and pre-filtering steps, the final dataset was subjected to supervised analyses using GeenSpring (Agilent Technologies) GEP and miR expression profile results were visualized by hierarchical clustering applying Ward’s method with Euclidean distance.

The microarray data have been submitted and will be available in Gene Expression Omnibus with the accession number GSE93795.

### Statistical analysis

Graphs and data analysis was carried out utilizing the PRISM software (version 6, GraphPad, Inc.). Where the means of two data sets were compared, significance was determined by a two-tailed Students t-test. Differences was considered significant at p < 0.05 (*p ≤ 0.05, **p ≤ 0.01, ***p ≤ 0.001). To evaluate the statistical significance of dose-response curves repeated-measures one-wayANOVA analysis was used.

## Electronic supplementary material

Supplementary Information
