# Supplementary material for: Common biological phenotypes characterize the acquisition of platinum-resistance in epithelial ovarian cancer cells
Source: Sci Rep. 2017 Aug 2;7:7104. doi: 10.1038/s41598-017-07005-1 (PMC5540908; doi:10.1038/s41598-017-07005-1)

Supplementary data

Common biological phenotypes characterize the acquisition of platinum-resistance in epithelial ovarian cancer cells

Maura Sonego[1], Ilenia Pellizzari[1], Alessandra Dall'Acqua[1], Eliana Pivetta[1], Ilaria Lorenzon[1], Sara Benevol[1], Riccardo Bomben[2], Paola Spessotto[1], Roberto Sorio[3], Valter Gattei[2], Barbara Belletti[1], Monica Schiappacassi[1] and Gustavo Baldassarre[1*].

[1] Division of Molecular Oncology, [2] Experimental OncoHematology and [3] Medical Oncology C CRO Aviano, IRCCS, National Cancer Institute, 33081 Aviano, Italy.

* To whom correspondence should be addressed:

Gustavo Baldassarre, MD
Division of Molecular Oncology
Centro di Riferimento Oncologico, National Cancer Institute
Via Franco Gallini, 2
33081 Aviano, Italy
Tel.: 39 0434 659 759/661
Fax: 39 0434 659 428
e-mail gbaldassarre@cro.it

Supplementary Figures

A

Cell lines	CDDP IC50	p53 mut	BRCA1 mut	BRCA2 mut
TOV-112D	4.7±1.03 μM	R175H (99.94%)	n.d.	n.d
MDAH	3.6±1.6 μM	R273H (99.09%)	n.d	ins1782fs (39.18%)
OVSAHO	3.6±0.9 μM	R342* (100%)	n.d	n.d
KURAMOCHI	4±1 μM	D281Y (99.59%)	n.d	R2318* (63.83%)

B

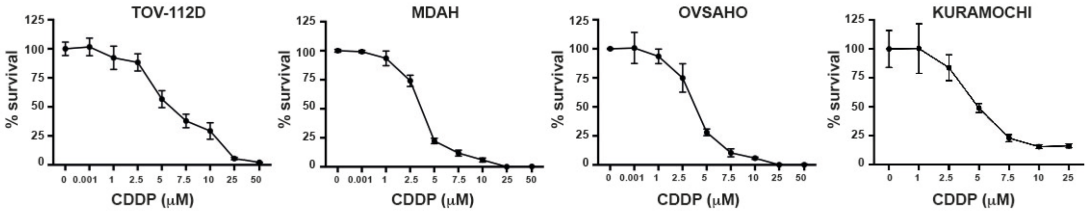

C

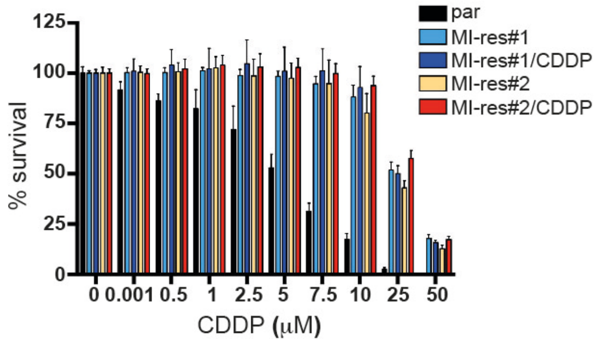

Supplementary Figure S1: Characteristics of the EOC cells used in this study.

(**A**) Table reporting the Cisplatin (CDDP) IC50 and the mutational status of TP53 and BRCA1 and BRCA2 genes in the EOC cell lines used in the study.

(**B**) Survival curves of parental EOC cells treated with cisplatin (CDDP) as indicated and used to evaluate the IC50.

(**C**) Dose-dependent survival curve of TOV-112D parental and MI-res cells treated with increasing concentration of cisplatin. MI-res cells were maintained for ≥ 2 moths in culture with (MI-res/CDDP) or without (MI-res) low dose of cisplatin (1μM) and then assessed in survival curve.

Results in **B** and **C** are expressed as the mean (±SD) of percentage of viable cells respect to untreated cells (n=3 biological replicates each performed in triplicate).

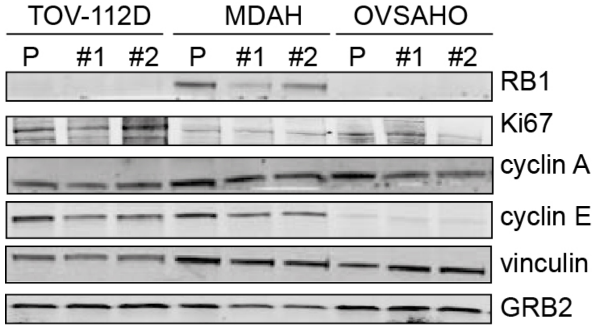

Supplementary Figure S2: MI-res cells showed an expression of proliferating markers similar to the one of their parental cells.

Western blot analyses evaluating the expression of Ki67 (expressed in proliferating cells), RB1 (expressed during G1) and Cyclin E and A (expressed in S and G2 phases of the cell cycle) in the indicated parental and MI-res cells cultured in exponentially growing conditions. The expression of Vinculin and GRB2 was used as loading control.

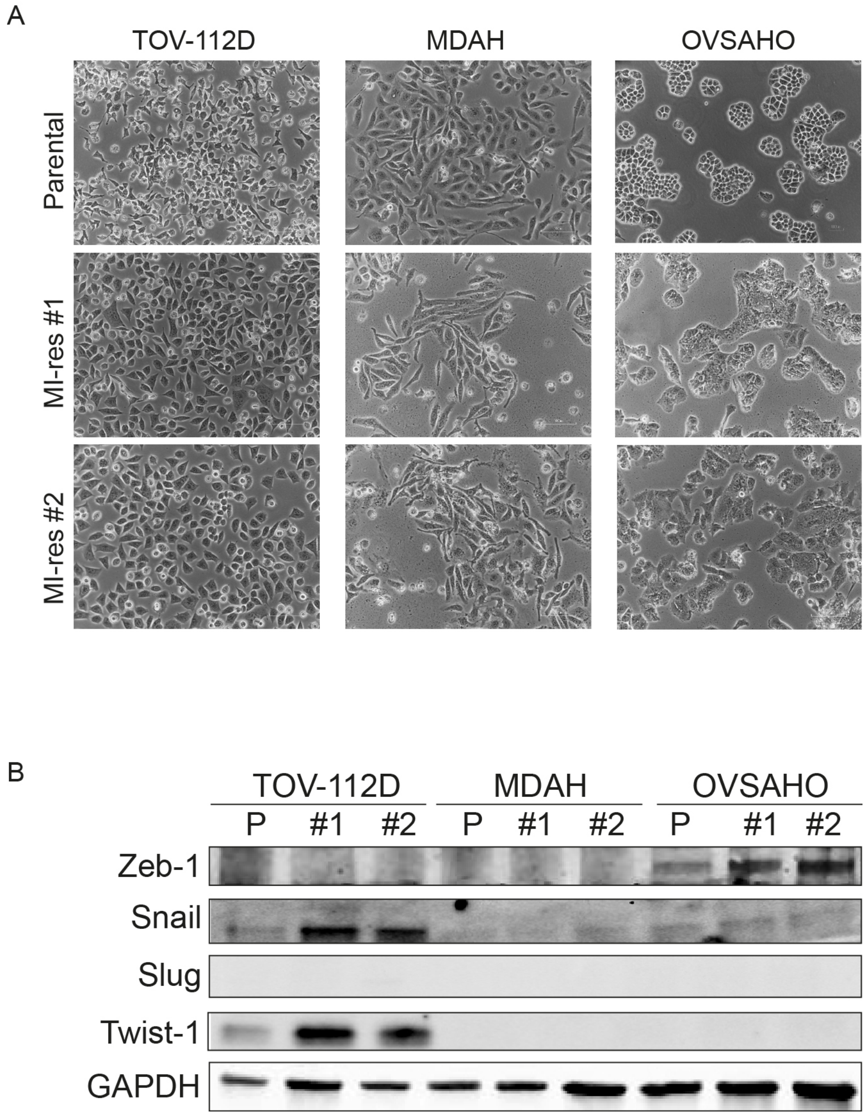

Supplementary Figure S3: Altered morphology is a common feature of MI-res cells. (**A**) Representative phase-contrast photomicrographs of EOC parental (top) and MI-res (middle and bottom) cells in exponential phase (20X, original magnification). (B) Western blot analyses of transcription factors involved in the regulation of the EMT process. GAPDH was used as loading control. P = parental cells. #1 and #2 indicate the two MI-Res clones.

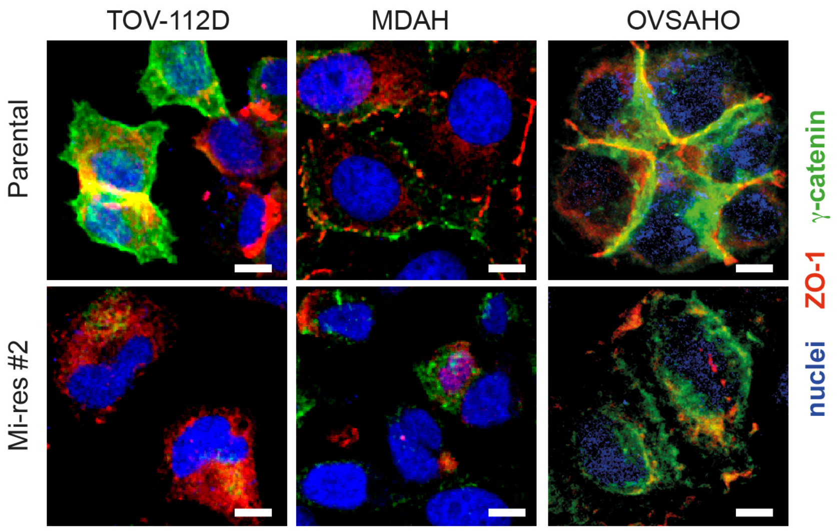

Supplementary Figure S4: MI-res cells failed to form well-organized cell-cell junctions. Representative images of γ-catenin (green) and ZO-1 (red) expression in parental and MI-res cells cultured in exponentially growing conditions. High magnification (63X objective 2X zoom) was used to better highlight cell-cell contacts in parental and MI-Res cells. Nuclei are pseudoclored in blue. Scale bar=11.5 µm.

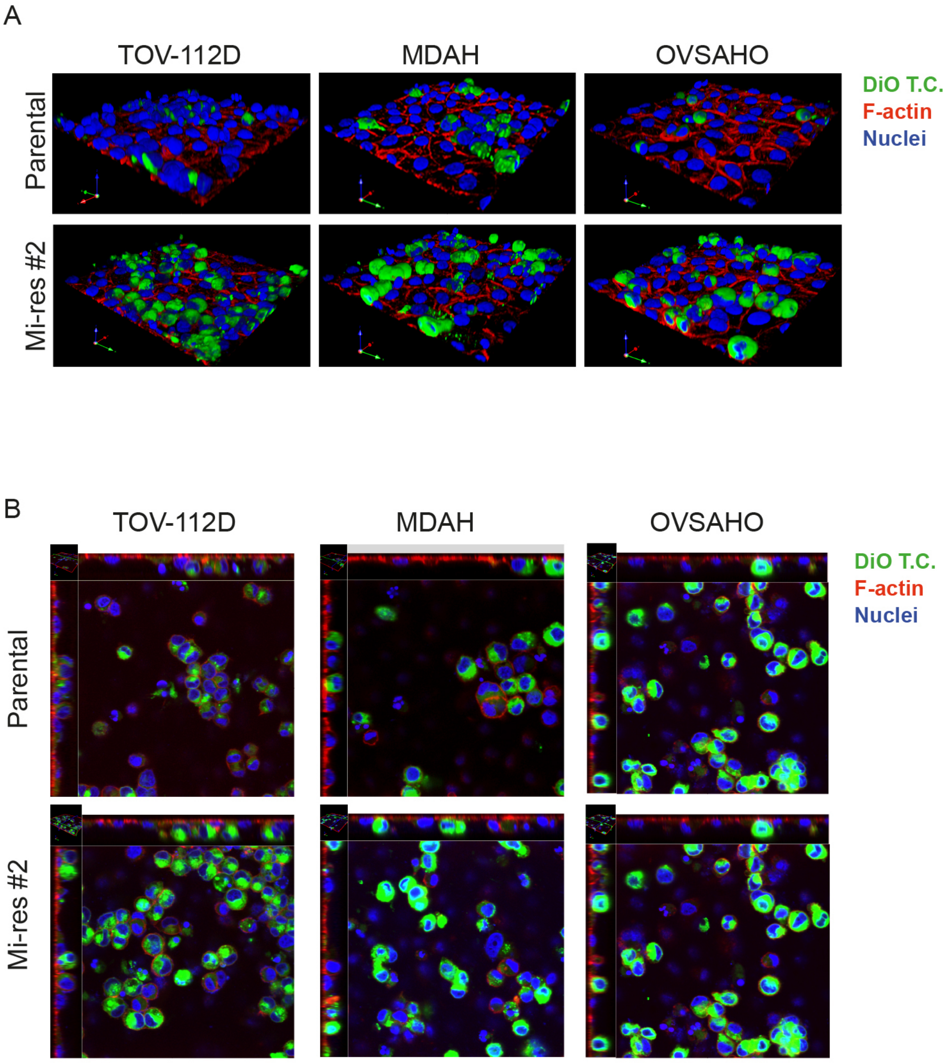

Supplementary Figure S5: MI-res cells better adhere to mesothelial cells.

(**A**) 3D reconstruction of confocal stack images of DiO-labeled parental and MI-res cells (green) cultured on a monolayer of mesothelial cells for 24 hours in the absence of serum. Cells were then fixed and stained with Phalloidin (F-Actin, red) and TO-PRO3 (nuclei, blue).

(**B**) Representative XY planes of confocal images of parental and MI-res cells labeled as in B. YZ and XZ projections show the contacts between mesothelial (well flat and delineated by red F-actin) and EOC cells ("spheric" and green labeled). **A** and **B** are projections of the images reported in Fig 3D.

A

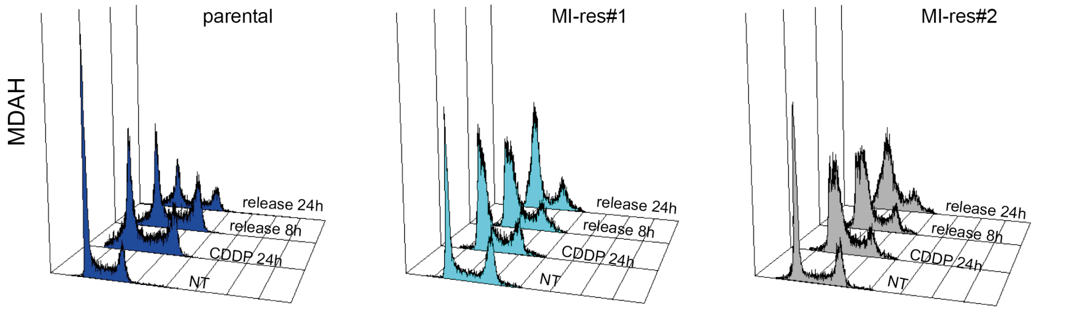

parental	% subG1 (±SD)	% G1 (±SD)	% S (±SD)	% G2/M (±SD)
NT	0.74±0.28	42.205±17	37.125±8.9	20.92±17.7
24h	9.97±2.5	26.51±0.87	46.03±0.35	17.18±1.97
+8h	8.87±1.12	15.65±0.81	57.29±4.74	19.17±3.66
+24h	10.42±0.62	17.7±1.25	48.61±12.1	16.76±1.31

MI-res1	% subG1 (±SD)	% G1 (±SD)	% S (±SD)	% G2/M (±SD)
NT	2.76±0.43	43.37±2.92	31.14±1.37	22.71±1.78
24h	2.78±0.95	22.42±6.99	63.89±1.23	12.65±3.94
+8h	1.99±0.42	19.15±3.10	63.28±1.99	13.49±4.06
+24h	3.6±2.08	7.07±1.64	72.57±0.76	13.50±0.18

MI-res2	% subG1 (±SD)	% G1 (±SD)	% S (±SD)	% G2/M (±SD)
NT	6.27±5.76	46.4±0.48	29.67±0.57	17.91±7.74
24h	3.36±0.27	17.9±0.52	65.26±1.33	11.47±0.93
+8h	3.27±0.95	16.42±0.22	65.75±0.01	12.40±0.95
+24h	6.29±2.28	9.89±1.68	69.85±3.39	11.06±1.44

B

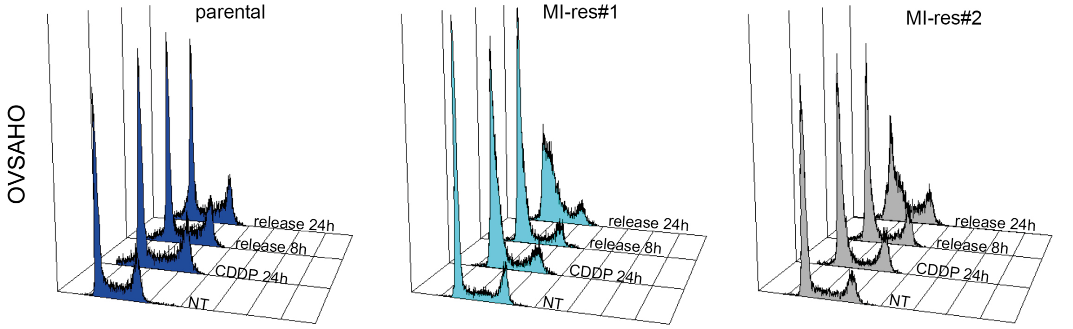

parental	% subG1 (±SD)	% G1 (±SD)	% S (±SD)	% G2/M (±SD)
NT	1.86±1.35	43.6±6.99	36.43±4.82	18.66±0.04
24h	6.71±0.07	41.09±4.6	31.66±5.01	21.18±0.08
+8h	6.65±1.44	41.22±5.12	31.39±5.27	21.7±1.52
+24h	11.82±1.53	38.44±4.43	32.03±4.65	18.48±1.51

MI-res1	% subG1 (±SD)	% G1 (±SD)	% S (±SD)	% G2/M (±SD)
NT	0.91±0.08	57.58±6.26	27.72±7.83	14.11±1.39
24h	1.06±0.04	43.23±4.62	39.06±4.26	10.28±0.78
+8h	1.71±1.29	47.42±6.6	37.35±1.57	10.31±2.11
+24h	4.96±2.99	23.51±8.22	57.03±9.39	9.54±0.72

MI-res2	% subG1 (±SD)	% G1 (±SD)	% S (±SD)	% G2/M (±SD)
NT	0.88±0.16	58.62±1.80	26.76±7.11	12.84±1.77
24h	2.47±0.48	44.83±5.76	36.85±1.20	10.93±1.83
+8h	2.98±0.05	45.48±2.47	40.72±3.24	11.19±0.91
+24h	5.98±0.18	22.29±6.6	57.53±0.52	9.3±0.98

Supplementary Figure S6: MI-res cells resolve faster the Cisplatin-induced cell cycle block respect to parental cells.

(**A-B**) FACS analyses of DNA content in MDAH (**A**) and OVSAHO (**B**) parental and MI-res cells untreated (NT) or treated with cisplatin (CDDP) for 24 hours and then released in cisplatin free medium (release) for additional 8 or 24 hours as indicated.

A representative histogram is shown for each cell line and the correspondent cell cycle distribution (mean ± SD n = 3 biological replicates) is reported in the lower tables.

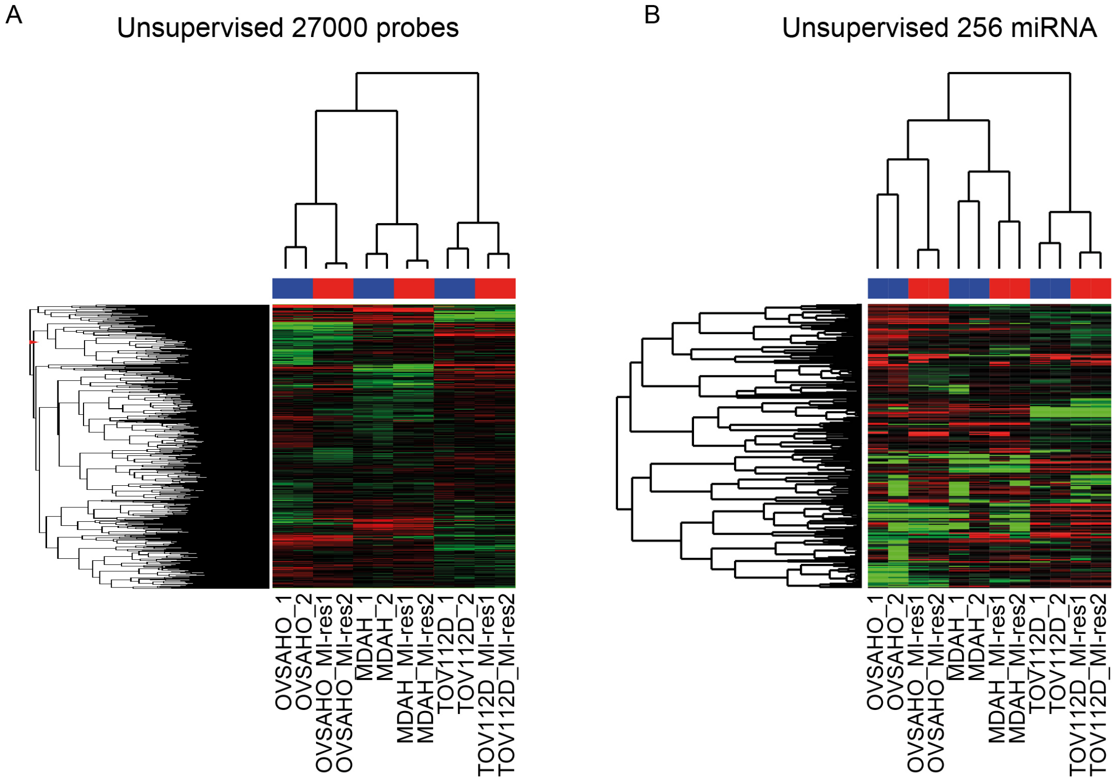

Supplementary Figure S7: Coding and non-coding genes expression in MI-res cells fails to identify common altered gene expression.

(**A-B**) Heat map of unsupervised clustering analyses evaluating the expression of coding and long non-coding RNAs (**A**) or microRNAs (**B**) in parental and MI-res cells as indicated. Unsupervised clustering demonstrated that parental and MI-res cells cluster together based on the cell type and not on the cisplatin resistance.

Primer	Sequence
MDR1 FW	TAATGCCGAACACATTGGAA
MDR1 REV	TCTTCACCTCCAGGCTCAGT
CTR1 FW	GGGGATGAGCTATATGGACTCC
CTR1 REV	TCACCAAACCGGAAAACAGTAG
CTR2 FW	ATACAGCGGTGCTTCTGTTTG
CTR2 REV	GGTTGGCAGGTTCACCAGTA
ATP-7A FW	TGACCCTAAACTACAGACTCCAA
ATP-7A REV	CGCCGTAACAGTCAGAAACAA
ATP-7B FW	ACCCGGAAGCACTGTAATTG
ATP-7B REB	TCTGAGCCTCTTCCACCAGT
SDHA FW	AGAAGCCCTTTGAGGAGCA-
SDHA REV	CGATTACGGGTCTATATTCCAG

Supplementary Table S1: Primers used in this study for qRT-PCR analyses.

Uncut Western Blots

Blots related to Supplementary Figure S2

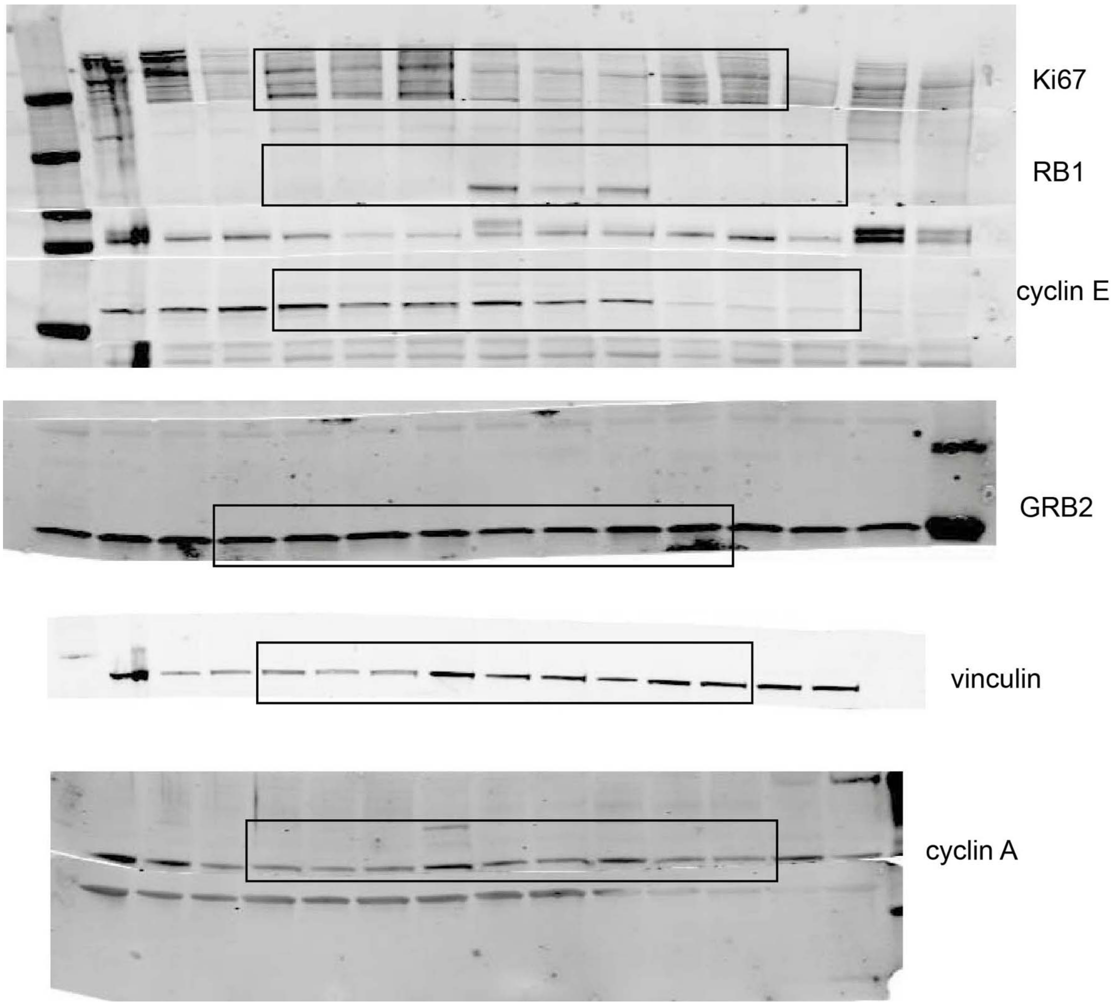

Blots related to Supplementary Figure S3B

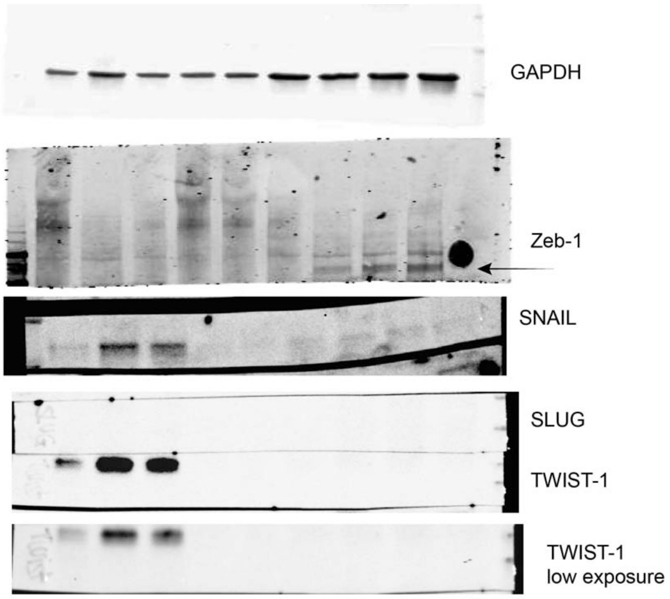

Blots related to Figure 4

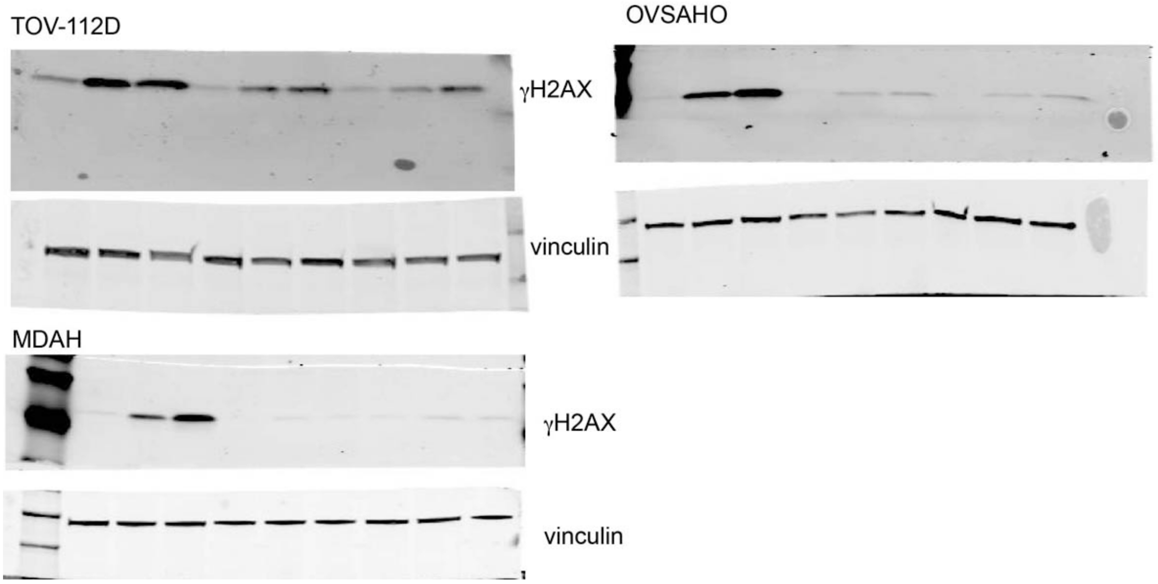

Supplement: Supplementary file 1 — Supplementary Information [file 41598_2017_7005_MOESM1_ESM.pdf]
